# Periodic cohort health examinations in the TAMRISK study show untoward increases in body mass index and blood pressure during 15 years of follow-up

**DOI:** 10.1186/1471-2458-12-654

**Published:** 2012-08-14

**Authors:** Tarja Kunnas, Kirsi Määttä, Pirjo Palmroos, Seppo T Nikkari

**Affiliations:** 1 Department of Medical Biochemistry, University of Tampere Medical School, Tampere, Finland; 2 Tullinkulma Occupational Health Unit, City of Tampere, Tampere, Finland

**Keywords:** Periodic health examinations, Body mass index, Hypertension

## Abstract

**Background:**

Obesity is a significant risk factor for hypertension and diabetes. A cohort of 50-year-old voluntary periodic health examination (PHE) participants was analyzed 15 years retrospectively. Our aim was to evaluate changes in body mass index (BMI) and blood pressure in subjects diagnosed with hypertension and/or diabetes in comparison with healthy controls.

**Methods:**

Voluntary periodic health examinations (PHE) of the citizens have been carried out by the city of Tampere, Finland. Health data, including body mass index (BMI) and blood pressure, were recorded every five years, starting at the age of 35 (baseline). A total of 339 subjects from the 50-year-old cohort having hypertension and/or diabetes were chosen to the study group. The control group included 604 subjects from the 50-year-old cohort who had the same follow-up information but were not diagnosed with hypertension and/or diabetes.

**Results:**

In the study group the mean BMI had increased from 26.1 at baseline to 28.5 at the final 15-year follow-up examination. The corresponding increase in the control group was from 23.8 at baseline to 25.5 at the final follow-up. The difference in change with time between the groups was statistically significant (p = 0.04). On the average, the controls gained 4.9 kilograms, whereas subjects in the study group gained 7.0 kilograms over the 15 years of follow-up. Systolic and diastolic blood pressures were also higher in the study group already at baseline and systolic blood pressure increased with time more in the study group than in the control group (p = 0.004).

**Conclusions:**

BMI and blood pressure were higher in the study group in comparison with the controls already at baseline at 35 years, and the differences were not favorably changed during the follow-up. Apparently, the effect of PHE had not been as efficient as planned on subjects in the study group, who were already slightly overweight at baseline.

## Background

Periodic health examinations (PHE) are endorsed as a means of preventive medicine and usually include medical history, physical examination and multiple screening using simple laboratory tests. Goals of PHE are early diagnosis of chronic diseases and the improving of patient understanding of health and disease [1].

PHE has been a fundamental part of medical practice for decades despite a lack of consensus on its value. In Canada, the roots of the annual PHE date back to 1861. In the 1970s and 1980s, both the Canadian Task Force on the Periodic Health Examination and the United States Preventive Services Task Force recommended abandoning the PHE in favor of regular appropriate evidence-based preventive care during regular visits [2,3]. A recent systematic review of medical databases suggests that the PHE may lessen patient worry, but other outcomes were vague [4]. It is clear that additional research is needed to assess the costs, benefits and harms as well as long-term outcomes, of the PHE [5].

Obesity is a significant risk factor for chronic diseases, such as hypertension and diabetes [6-8]. More than 50% of European adults are obese or overweight [9]. We evaluated changes in body mass index (BMI) and blood pressure during 15 years of PHE follow-up. Subjects diagnosed with hypertension and/or diabetes by the age of 50 were compared with controls.

## Methods

The Tampere adult population cardiovascular risk study (TAMRISK) is a prospective, longitudinal population based health survey study in Tampere, a city in southern Finland with a population of 210 000 [10,11].

The Tampere city health care centre has provided regular PHEs, for screening and counselling, for the adult population of the city since 1980. All 40- and 50-year-old inhabitants have been invited to participate in these health surveys. During some periods, also 35- and 45-year-olds have been invited to participate. The PHE consisted of one 60-minute session with a public health nurse at the centre's health examination unit. In the session, the questionnaire information and screening tests were reviewed. Counseling was given on topics selected by the participant and also on findings of the screening tests.

Information is available for some participants for 15 years of consecutive 5-year follow-ups concerning risk factors for cardiovascular diseases, including family history, blood pressure, lipid values, smoking, waist circumference, diabetes exercise and eating habits. Also data on diseases has been recorded. Presently, invitations for the survey are sent to all subjects from Tampere that are 40 or 50 years old that year.

All subjects who had a self-reported diagnosis of hypertension and/or diabetes (made by a physician, n = 681) were chosen from a PHE 50-year-old cohort (n = 6000). Those subjects who also had information on 35-, 40-, and 45- year PHE formed the study group (n = 339). The control group included subjects from the same 50-year-old cohort who were apparently healthy, and had the same follow-up information. Controls were chosen for every subject in the study group by matching them according to sex (n = 604). Baseline clinical examinations had taken place during the calendar year when the subject caught the age of 35 in 1988–91. At the age of 40 the same subjects were invited to health examination again if they still lived in Tampere. This happened in 1993–96. The third follow up took place in 1998–2001 at the age of 45. The last follow up was in years 2003–06 at the age of 50. The follow-up time was 15 years. The Ethics Committees of the Tampere University Hospital and the City of Tampere approved the study. Written informed consent for participation in the study was obtained from all participants.

### Baseline measurements

The basic evaluation in 1988–91 included an interview by a public health nurse. The interview was conducted using a structured questionnaire about health and health-related behavior, including questions about current and previous diseases. Information on current and previous diseases was based on self-report of diagnosis by a physician, including history of MI and diabetes. The questionnaire also assessed symptoms and ailments experienced within the past six months. These included questions of health in general and mental health. Questions of health-related behavior included current and past smoking. The frequency of physical exercise comprised both leisure and commute related activity. Quantitative estimation of the alcohol intake was carried out by using three structured questions to determine the amount and frequency of drinking. The total mean consumption of all alcoholic drinks was used, expressed as grams of pure ethanol per week. Physical examination included a single blood pressure (BP) measurement (mm of mercury) using a calibrated mercury sphygmomanometer. Serum total cholesterol (mmoles/liter) was measured by enzymatic techniques. Height (cm) and weight (kg) were recorded from which the body mass index was calculated.

### Statistical analyses

The description of data was made by means and standard deviations for continuous variables and by proportions for categorical variables. The descriptions were constructed separately to the study group and controls. The differences in dichotomous variables were compared with Fisher's exact test, in other categorical variables with Chi-square test and in continuous variables with *t*-test, or Mann Whitney *U* test if the distribution was skewed. The analysis of variance for repeated measures was used to assess the differences in changes of body mass index and blood pressure between the study group and the control group during the follow up time. Normality of the distributions of the dependent variables were tested using Kolmogorov-Smirnov test, and logarithmic transformation was applied if the distribution of the variable was skewed. The changes were calculated in relation to the baseline measurement at the age of 40, 45 and 50. The model included the main effects of group factor and time, and their interaction. The baseline measurement of dependent variable was included in the model as a covariate. The risk level in the analyses was set equal to p-value of 0.05. Analyses were made using SPSS for Windows (version 16.0) program.

## Results

The characteristics of the 339 cases belonging to the now 50-year-old study group with diagnosed hypertension (91%), diabetes (15%) or both (9%), and their 604 controls are shown in Table 1 at baseline, when they were 35 years old. At the age of 35, subjects in the study group had already higher mean BMI, systolic and diastolic blood pressure and serum cholesterol as compared to controls. Physical exercise and daily smoking did not differ between the groups at the age of 35 or during the whole follow-up. In the study group the self-reported health status was poorer and more alcohol was used per week compared to controls at the age of 35. This difference persisted also at the age of 50 (data not shown). In the study group there were already 9 subjects with diagnosed hypertension and two with diabetes at the age of 35. Subsequently, the mean BMI increased from 26.1 at baseline to 28.5 at the last follow-up examination in the study group, whereas in the 604 controls the corresponding increase was from 23.8 at baseline to 25.5 at the last follow-up. On average, controls gained 4.9 kilograms, whereas subjects with hypertension or diabetes at the age of 50 gained 7.0 kilograms over the 15 years of follow-up.

**Table 1:** Background characteristics of subjects examined at baseline in 1988–91 at the age of 35 stratified by whether or not they had hypertension or diabetes (as diagnosed by a physician) by the age of 50

	**Study group**	**Controls**	p*
N	339	604	
Male gender (%)	62	61	0.726
Married (%)	82	83	0.647
In working life (%)	96	93	0.207
Physical exercise at least 3 times/week (%)	25	28	0.442
Current daily smokers (%)	33	28	0.226
Self-reported health status at least quite good (%)	69	80	0.004
Alcohol consumption as grams/week (SD)	95.3 (96.2)	79.2 (90.0)	0.047
Height (cm), mean (SD)	172.8 (9.5)	172.8 (9.2)	0.910
BMI (kg/m^2^), mean (SD)	26.1 (4.4)	23.8 (3.2)	<0.001
Diastolic BP (mm Hg), mean (SD)	86 (10)	78 (8)	<0.001
Systolic BP (mm Hg), mean (SD)	135 (13)	124 (10)	<0.001
Hypertension (%)	9	0	<0.001
Diabetes (%)	2	0	0.016
Serum cholesterol (mmol/l), mean (SD)	5.5 (1.0)	5.2 (1.0)	0.001

*Fisher’s test for categorized variables, Mann Whitney *U* test for continuous variables. BP, blood pressure; BMI, body mass index.

**Figure 1:**
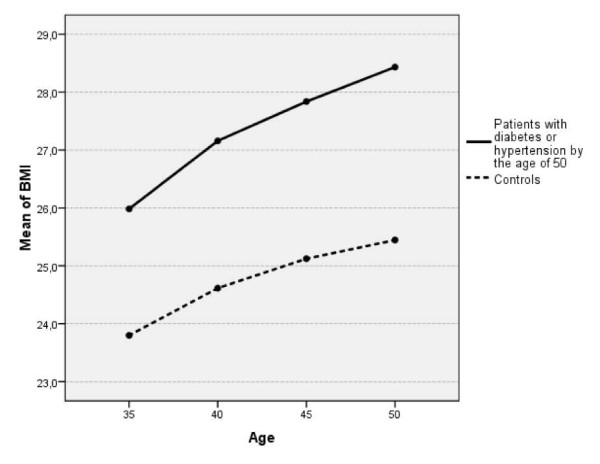
The development of body mass index (BMI) in the study group and controls during the time the periodic health examinations were conducted.

The mean changes in BMI and blood pressure are shown in Table 2. We found a statistically significant interaction in the change of BMI between the study group and the control group, and the follow-up time (p = 0.04). The BMI increased more in the study group than in the control group, and the increase rate was constant over the follow-up time. In the control group BMI also increased, but the increase rate was lower especially during the last 5 years of the follow-up (Figure 1).

**Table 2:** The change in body mass index (BMI) and blood pressure in subjects from the TAMRISK study stratified by whether or not they had hypertension or diabetes by the age of 50

	**Study group**	**Controls**	**P-value (interaction in change between group and time effects)**	**Difference (95% CI)**	**P-value (*t*-test)**
Change in BMI (kg/m^2^)			0.04		
40 years of age	0.9 (2.3)	0.4 (1.8)		0.43 (0.14;0.72)	0.008
45 years of age	1.8 (2.4)	1.3 (1.8)		0.59 (0.30;0.87)	<0.001
50 years of age	2.4 (3.1)	1.7 (2.2)		0.73 (0.36;1.11)	<0.001
Change in systolic blood pressure (mmHg)			0.004		
40 years of age	2.5 (10.5)	0.6 (8.2)		1.95 (0.54;3.36)	0.007
45 years of age	6.2 (15.5)	2.2 (10.7)		3.99 (1.96;6.03)	<0.001
50 years of age	6.2 (20.4)	5.3 (14.1)		0.90 (−1.73;3.53)	0.501
Change in diastolic blood pressure (mmHg)			0.06		
40 years of age	2.6 (10.0)	1.4 (7.1)		1.17 (−0.14;2.48)	0.080
45 years of age	4.5 (11.6)	2.4 (7.4)		2.08 (0.58;3.57)	0.007
50 years of age	5.6 (14.0)	5.7 (9.2)		−0.16 (−1.95;1.63)	0.859

The change was calculated in relation to baseline (at 35 years of age) during the 15-year follow-up when the periodic health examinations were conducted. Positive values reflect the increase in BMI or blood pressure. Values are means (standard deviations).

A statistically significant interaction was found in the change of systolic blood pressure between the study group and the controls, and the follow-up time (p = 0.004). The increase was slightly higher in the study group than in the control group. In the study group the highest increase was found between 40 and 45 years of age, whereas in the control group the increase rate was at the highest between the age of 45 and 50.

The diastolic blood pressure remained higher in the study group than in the control group during the follow-up time, but the interaction in the change between the group and the time effects was not statistically significant (p = 0.06). At the age of 50, a total of 61% of the study group were on self-reported blood pressure medication.

## Discussion

A difference was found in the change of BMI between the study group and the control group during the follow-up time. In the study group the increase rate was higher and it was constant over the follow-up time, whereas in the control group the rate slowed slightly during the last 5 years of the follow-up. This suggests that the effects of the PHE had not been efficient enough to stop the weight gain in either group. Especially in the study group the remarkable increase in BMI was seen despite the PHE conducted during the follow-up time and there were no signals of weight gain slowing down.

The subjects in the study group had a higher level of BMI at baseline. Because obesity in known to be a major risk factor for cardiovascular disease and diabetes, the subjects in the study group were in greater risk already on that basis compared with the control group at the very beginning. In that sense it would have been possible to allocate the preventive actions to these subjects in the PHEs. The result suggests that PHE could not intervene with the weight gain of subjects who had a high risk to cardiovascular disease and diabetes.

The increase in blood pressure was in high extent consistent with that of BMI. The blood pressure (both systolic and diastolic) was at higher level in the study group than in the control group already at baseline. The increase in systolic blood pressure was also greater in the study group than in the control group between the ages of 40 and 45. Favorable effects of PHE were not seen in relation to changes in blood pressure, even though blood pressure medication had been started for more than half of the subjects in the study group.

Participant views of the PHE in Tampere have been previously examined in 1995, in a 45-year-old cohort [12]. According to the participants' accounts, the discussions and counseling with a public health nurse had dealt with living habits and, to a lesser extent, with diseases, symptoms and the prevention of illness. Virtually all respondents saw the PHE program as beneficial for everyone. Opinions were divided on its benefits compared with medical help from a physician. Looking at the present results, the PHE, conducted at 5-year intervals by a public health nurse, had not been very effective in intervening with living habits, since BMI continued to increase in the study group, who were already at risk at the age of 35 years. It has been reported that adults who were overweight but not obese (i.e., 25.0 ≤ BMI ≤ 29.9) were at significantly increased risk of developing numerous health conditions, such as diabetes, gallstones, hypertension, heart disease, and stroke [6]. Although the study group had a diagnosis of diabetes or hypertension by a physician, their medical intervention was not sufficient in terms of blood pressure medication. Certainly a larger proportion of cases would have been in need for more regular intervention and medication.

At the age of 35, subjects in the study group used more alcohol per week (95 g) than controls (79 g). This alcohol consumption should be considered in the context that self-reporting of drinking is commonly unreliable and the official per capita mean consumption of absolute ethanol in Finland for inhabitants 15 years and older is 8.4 l per year, corresponding to 162 g per week [13]. Since physical activity was not different between the study group and controls, the difference in alcohol intake has potential to explain the remarkable increase of BMI in the study group compared to controls during the follow-up time.

The strength of present study is the large cohort from which the data was collected. The data analyzed included 943 subjects all born in the four year period 1951–54 and followed up for 15 years. Thus there was no confounding by age. The 15-year follow-up of the cohort enables to study trends in health factors. However, since the study group was restricted to residents of a large city in Finland poses a challenge to how broadly one can apply the findings. Because the studied population represented only Caucasians, our findings cannot be generalized to minority groups.

## Conclusions

The unfavorable differences in BMI and blood pressure between the study group, who had hypertension and/or diabetes at the age of 50, and the controls were seen already at the 35-year-old PHE and they were not corrected during the 15-year follow-up time when PHE were conducted. This suggests that the effect of PHE had not been as favorable as desired among the subjects in the study group, who were at risk already at the age of 35.

## Competing interests

The authors declare that they have no competing interests.

## Authors’ contributions

TK, KM, PP and SN had substantial contributions to conception and design and interpretation of data and writing the manuscript. All authors read and approved the final manuscript.

## Pre-publication history

The pre-publication history for this paper can be accessed here:

http://www.biomedcentral.com/1471-2458/12/654/prepub
